# Modulating neuroinflammation and cognitive function in postoperative cognitive dysfunction via CCR5‐GPCRs‐Ras‐MAPK pathway targeting with microglial EVs

**DOI:** 10.1111/cns.14924

**Published:** 2024-08-14

**Authors:** Zheng Qi, Junlin Peng, Haitao Wang, Li Wang, Yu Su, Lan Ding, Bin Cao, Yingying Zhao, Qinghe Xing, Jian‐jun Yang

**Affiliations:** ^1^ Department of Anesthesiology, Pain and Perioperative Medicine The First Affiliated Hospital of Zhengzhou University Zhengzhou China; ^2^ Biobank of The First Affiliated Hospital of Zhengzhou University Zhengzhou China; ^3^ Institutes of Biomedical Sciences Fudan University Shanghai China; ^4^ Department of Neurology The First Affiliated Hospital of Zhengzhou University Zhengzhou China

**Keywords:** CCR5, extracellular vesicles, neuroinflammation, postoperative cognitive dysfunction

## Abstract

**Aims:**

Postoperative cognitive dysfunction (POCD) is prevalent among the elderly, characterized primarily by cognitive decline after surgery. This study aims to explore how extracellular vesicles (EVs) derived from BV2 microglial cells, with and without the C‐C chemokine receptor type 5 (CCR5), affect neuroinflammation, neuronal integrity, and cognitive function in a POCD mouse model.

**Methods:**

We collected EVs from LPS‐stimulated BV2 cells expressing CCR5 (EVs^M1^) and from BV2 cells with CCR5 knockdown (EVs^M1‐CCR5^). These were administered to POCD‐induced mice. Protein interactions between CCR5, G‐protein‐coupled receptors (GPCRs), and Ras were analyzed using structure‐based docking and co‐immunoprecipitation (Co‐IP). We assessed the phosphorylation of p38 and Erk, the expression of synaptic proteins PSD95 and MAP2, and conducted Morris Water Maze tests to evaluate cognitive function.

**Results:**

Structure‐based docking and Co‐IP confirmed interactions between CCR5, GPR, and Ras, suggesting a CCR5‐GPCRs‐Ras‐MAPK pathway involvement in neuroinflammation. EVs^M1^ heightened neuroinflammation, reduced synaptic integrity, and impaired cognitive function in POCD mice. In contrast, EVs^M1‐CCR5^ reduced neuroinflammatory markers, preserved synaptic proteins, enhanced dendritic spine structure, and improved cognitive outcomes.

**Conclusion:**

EVs^M1^ induced neuroinflammation via the CCR5‐GPCRs‐Ras‐MAPK pathway, with EVs^M1‐CCR5^ showing protective effects on POCD progression, suggesting a new therapeutic strategy for POCD management via targeted modification of microglial EVs.

## Graphical abstract

Administer anesthesia and perform surgery to promote M1 polarization of microglia and the release of EVs^M1^ with high CCR5 expression. EVs^M1^ promotes CCR5 expression on the neuronal surface, which triggered activation of the GPCRs‐Ras‐Mapk signaling pathway by the binding of CCL3/4/5 to their receptor CCR5 on neurons, causing neuronal degeneration, synaptic loss, and ultimately POCD.

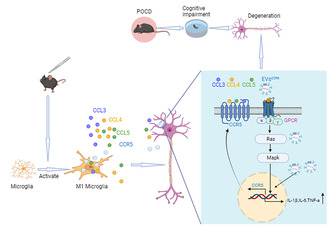

## 1 INTRODUCTION

Postoperative cognitive dysfunction (POCD) is a complex neurocognitive disorder affecting many elderly patients following surgical procedures, characterized by a decline in memory, attention, and executive functions.^1^ This decline significantly impacts the quality of life and increase the risk of long‐term cognitive impairments. While the complete pathophysiology of POCD is not fully understood,^2^ emerging evidence suggests that neuroinflammation plays a pivotal role in its development.^3^

Neuroinflammation, characterized by the activation of microglial cells—the central nervous system's resident immune cells,^4^, ^5^ is a key factor in the neurodegenerative processes associated with POCD.^6^ Upon activation, microglia transition into a pro‐inflammatory state (M1 phenotype), releasing cytokines and chemokines such as IL‐1β, IL‐6, and TNF‐α, which contribute to neuronal damage and cognitive decline.^7^, ^8^

The C‐C chemokine receptor type 5 (CCR5), expressed on neurons and microglial cells, has been identified as a significant mediator of inflammatory responses within the brain.^9^, ^10^ CCR5 and its ligands are involved in various central nervous system (CNS) pathologies, where they influence cell signaling pathways promoting inflammation and neuronal damage. Notably, the interaction between CCR5 and its ligands activates the MAPK pathway, leading to the production of inflammatory cytokines such as CCL3, CCL4, and CCL5, crucial in the progression of neuroinflammatory and neurodegenerative diseases.^11^, ^12^

G‐protein‐coupled receptors (GPCRs) are essential mediators of cellular signal transduction and play a crucial role in various physiological and pathological processes, including neuroinflammation.^13^ Upon activation by extracellular ligands, GPCRs activate Ras, a small GTPase that acts as a molecular switch. This activation initiates the MAPK signaling cascade, involving the sequential phosphorylation and activation of Raf, MEK, and ERK kinases.^14^ Recent studies have highlighted the involvement of the Ras‐MAPK pathway in neuroinflammatory processes, suggesting its potential as a therapeutic target for neurodegenerative conditions.^15^

Extracellular vesicles (EVs) are nanoscale vesicles released by cells, which include apoptotic vesicles, exosomes, and microvesicles. EVs typically range in size from 30 to 5000 nm and consist of proteins, nucleic acids, and other compounds crucial for intercellular communication.^16^ Several studies have outlined the functions of EVs in the development, diagnosis, and therapy of neurological illnesses.^17^ The role of EVs in mediating communication between cells in the CNS offers a novel perspective on modulating neuroinflammatory responses.^18^, ^19^, ^20^ EVs derived from microglial cells can carry a range of bioactive molecules, including inflammatory mediators, influencing neuronal function and survival.^21^

In this study, we hypothesized that altering the expression of CCR5 on activated microglial (BV2^M1^)‐derived EVs (EVs^M1^) can modulate their inflammatory potential, and thereby, influence the course of POCD. We investigated the effects of EVs^M1^ with and without CCR5, on neuronal inflammation, apoptosis, and cognitive function in a mouse model of POCD. By exploring the interplay between microglial activation, CCR5 signaling, and neuronal damage, our study aims to uncover potential therapeutic targets that could alleviate or prevent the onset of cognitive impairments following surgical interventions. This research could provide a foundation for developing strategies to modulate the inflammatory milieu in the CNS, offering approach avenues for the treatment of POCD and related neurocognitive disorders.

## 2 MATERIALS AND METHODS

### 2.1 Animals

Twenty‐month‐old male C57BL/6 mice were obtained from the Animal Center of Zhengzhou University, Zhengzhou, China, and maintained under a 12‐h light/dark cycle with free access to food and water, room temperature (22 ± 1°C) and 50 ± 10% humidity. The study protocol was approved by the Ethics Committee of the first affiliated Hospital of Zhengzhou University (2021‐KY‐0771‐002), and all procedures were performed in accordance with the Guidelines for the Care and Use of Laboratory Animals by the National Institutes of Health (Bethesda, MD, USA).

### 2.2 Establishment of a POCD mouse model

Each group of mice was assigned a permanent numerical designation in the cages after being randomly divided into groups. An exploratory laparotomy was conducted under anesthesia in a chamber containing 1.5% oxygen in isoflurane under sterile circumstances, as detailed in our prior study.^22^ The belly was shaved and cleansed with povidone‐iodine. A 1 cm incision was made in the middle of the abdomen to explore the internal organs, intestines, and muscles. The abdominal cavity lining and skin were sutured closed using sterile 4–0 chromic gut sutures. The wound was treated with polysporin (Pfizer, USA) to avoid infection. The complete surgical procedure was conducted under isoflurane anesthesia and had a duration of 10 min.

### 2.3 Microglia culture and LPS stimulation

BV2 microglial cells (#CL‐0493, Procell, China) were cultured in Dulbecco's Modified Eagle Medium (DMEM) supplemented with 10% fetal bovine serum and 1% penicillin–streptomycin (Gibco, ThermoFisher, USA). Cells were stimulated with 2 μg/mL lipopolysaccharide (LPS, #L4391, Sigma‐Aldrich, St. Louis, MO, USA) for 48 h to induce activation.

### 2.4 siRNA knockdown

The CCR5 siRNA oligonucleotides and siRNA controls were purchased from Promega in Shanghai, China. Transfected 2 × 10^5^ LPS‐treated BV2 cells with a final concentration of 10 μM CCR5 siRNA and siRNA controls using lipofectamine RNAiMAX transfection reagent (#11668019, Invitrogen, USA). Validated mRNA level change at 24‐h post‐transfection using qRT‐PCR with primers CCR5‐Fw (5′‐ATGATTCCTGGGAGAGACGC‐3′) and CCR5‐Rev (5′‐AGCCAGGACGGTCACCTT‐3′), normalized to GAPDH.

### 2.5 Isolation of extracellular vesicles

BV2 (2 × 10^6^) microglia were seeded in T175 flasks (#431080; Corning, USA) with Dulbecco's Modified Eagle Medium (DMEM) supplemented with 10% fetal bovine serum and 1% penicillin–streptomycin (Gibco, ThermoFisher, USA). After cell confluency reached 80%, medium was changed to DMEM supplemented with 1% penicillin–streptomycin. Supernatant was harvested after 48 h incubation, and then EVs were isolated using differential centrifugation and ultracentrifugation stages, following our established protocols.^23^ We progressively centrifuged the microglia at 300×*g* for 10 min to eliminate cells, then at 2000×*g* for 10 min to remove dead cells, and finally at 10,000×*g* for 30 min to eliminate cell debris. We then ultracentrifuged the supernatant at 120,000×*g* for 70 min at 4°C, and then resuspended the pellet in PBS. The solution was passed through a 0.22 μm filter, the extracellular vesicles were collected by centrifugation at 120,000×*g* for 70 min at 4°C, and the resulting pellet was suspended in 200 μL of PBS and kept at −80°C for future use in studies.

### 2.6 EVs labeling and uptake identification using PKH26

EVs were labeled with PKH26 (Sigma‐Aldrich, St. Louis; USA) as per manufacturer's guidelines. Initially, 100–200 μL of fresh EVs samples (and 200 μL PBS, as negative control) were mixed with 500 μL Diluent C and 6 μL PKH26, stirred for 30 s, and incubated for 5 min in darkness at room temperature. To halt staining, 2 mL of 10% BSA in PBS was added, followed by a 70‐min ultracentrifugation at 120,000×*g* at 4°C to remove excess dye and isolate PKH26‐labeled EVs, which were then stored at −80°C. For uptake analysis, 2 μL of the labeled EVs were stereotactically injected into mice. EVs internalization analysis in vivo and in vitro was performed using an IVIS imaging system and fluorescent microscopy, respectively.

### 2.7 Transmission electron microscopy

For transmission electron microscopy (TEM), freshly extracted EVs were suspended again in cold phosphate‐buffered saline (PBS). For negative staining, 20 μL of the EVs solution was applied to copper grids with a carbon coating and left for 5 min at room temperature. The grid was treated with 2% phosphotungstic acid (Servicebio, China) for 1 min and then dried with filter paper at room temperature. The EVs were examined using a Transmission Electron Microscope (#HT7800, Hitachi, Japan) with an acceleration voltage of 100 kV and 100,000 times magnification.

### 2.8 Nanoparticle tracking analysis

Nanoparticle tracking analysis (NTA) was utilized to assess the distribution of particle size and concentration in EVs preparations with a ZetaView instrument (Particle Metrix, Germany). NTA accuracy was validated using 100 nm polystyrene beads (Sigma‐Aldrich, Germany) just before doing the tests. The EVs samples were diluted at a ratio of 1:100 in PBS and measurements were taken at 25°C. Five measurements of 30 s each were recorded for each EVs sample.

### 2.9 Structure‐based protein interaction interface analysis between CCR5, GPCRs (GPRα, GPRβ, GPRγ), and Ras

Structure of CCR5 (ID:4Mbs), GPCRs (ID:8Kh4), and Ras (ID:1LxD) was downloaded from the PDB database (https://www.rcsb.org/). Prediction of potential interaction interface between CCR5, GPCRs, and Ras was obtained from ZDOCK server (https://zdock.wenglab.org/). Prediction results were visualized using the PyMol tool (https://pymol.org).

### 2.10 Co‐Immunoprecipitation (Co‐IP)

Co‐IP was performed using a Co‐IP kit (#88804, ThermoFisher, USA) following the manufacturer's manual. Lysates were precleared with protein A/G agarose beads and incubated overnight with primary antibodies against CCR5, GPR (GPRα, GPRβ, and GPRγ), and Ras. The antibody–protein complexes were captured with fresh beads, and separated via SDS‐PAGE and transferred onto PVDF membranes. Western blotting was performed using specific primary antibodies followed by HRP‐conjugated secondary antibodies.

### 2.11 Western blot

Protein extraction was performed using RIPA buffer (ThermoFisher, USA) containing protease and phosphatase inhibitors (#04906845001, Roche Diagnostics, USA). Protein concentrations were measured using a BCA Protein Assay Kit (#23225, Pierce, ThermoFisher, USA). Proteins were separated on 12% SDS‐PAGE, transferred to a 0.22 μm PVDF membranes (Bio‐Rad, Hercules, CA, USA), and subsequently blocked with 5% BSA in TBST for 1 h at room temperature. The membranes were treated overnight with the primary antibodies: CD9 (EVs'‐positive biomarker, #10626D, ThermoFisher) at a dilution of 1:1000, CD63 (EVs'‐positive biomarker, #10628D, ThermoFisher) at a dilution of 1:1000, GM130 (EVs'‐negative biomarker #12480 T, Cell Signaling Technology, USA) at a dilution of 1:1000, CCR5 (#PA5‐78950, ThermoFisher) at a dilution of 1:2000, p38 MAPK (#9212, Cell Signaling Technology, USA) at a dilution of 1:1000, Phospho‐p38 MAPK(#4511, Cell Signaling Technology, USA) at a dilution of 1:1000, p44/42 MAPK(#9102, Cell Signaling Technology, USA) at a dilution of 1:1000, and Phospho‐p44/42 MAPK (#4370, Cell Signaling Technology, USA) at a dilution of 1:1000 4°C overnight, followed by 1 h incubation with secondary antibody (Jackson Immuno Research) at room temperature. Detection was conducted using an ECL system (ThermoFisher, USA). The band density was evaluated with ImageJ software version 1.8.0 (National Institutes of Health in Bethesda, MD, USA).

### 2.12 Immunofluorescence

The brain samples were immersed in 4% paraformaldehyde for 24 h, then encased in paraffin wax, and sliced into slices that were 6 μm thick. The sections were deparaffinized using Histol (#6640.1, ROTH), followed by progressive rinsing in varying concentrations of pure ethanol (100%, 96%, 70%, and 50%), and subsequently blocked with 1% BSA. Brain samples or cells were exposed to specific primary antibodies (Iba1, 1:300; MAP2, 1:250; SYN, 1:200; PSD95, 1:200; p‐Erk, 1:200; p‐p38, 1:200) overnight at 4°C. They were then rinsed with PBS and treated with corresponding secondary antibodies (Alexa Fluor® 488 Conjugate, #4412, Cell signaling or Alexa Fluor® 594 Conjugate, #8889, Cell signaling). The specimens were stained with DAPI (#D3571, ThermoFisher, USA) for 15 min in the dark and then observed using a fluorescence microscope. The fluorescence intensity of each sample was measured in three randomly chosen regions using ImageJ software developed by the National Institutes of Health in the United States.

### 2.13 Golgi Staining

Brain sections were stained using the FD Rapid GolgiStain Kit (FD NeuroTechnologies, Columbia, MD, USA, Catalog #PK401) following the manufacturer's instructions to visualize neuronal dendritic spines.

### 2.14 Quantitative Real‐Time PCR (qRT‐PCR)

Total RNA was extracted using the RNeasy Mini Kit (#74104, Qiagen, Germany). cDNA synthesis was performed with the High‐Capacity cDNA Reverse Transcription Kit (#4368814, Applied Biosystems, CA, USA). qRT‐PCR was conducted using SYBR Green PCR Master Mix (#4309155, Applied Biosystems, United Kingdom) on a StepOnePlus Real‐Time PCR System (Applied Biosystems, United Kingdom). Gene expression was normalized to GAPDH.

### 2.15 ELISA

Concentrations of CCL/3/4/5 in cell culture supernatants were quantified using ELISA kits according to the manufacturer's protocols (R&D Systems, Minneapolis, MN, USA).

### 2.16 Fluoro‐Jade B (FJB) Staining

Brain sections were stained with Fluoro‐Jade B (#TR‐150‐FJB, Biosensis, Thebarton, Australia) to identify degenerating neurons, following the manufacturer's instructions as previously described.^22^

### 2.17 Morris Water Maze (MWM) Test

Mice were acclimated to the laboratory for 24 h prior to testing in a round black stainless steel pool with four quadrants and a hidden circular platform (10 cm diameter), submerged 1–2 cm below the water surface dyed to obscure the platform. Water was maintained at 22°C–24°C. During training, mice were released from different quadrants, with their paths and latency to find the platform monitored by an automatic system. If a mouse did not locate the platform within 60 s, it was guided there, allowed to rest for 10 s, and then removed and dried. Each mouse underwent four daily trials with 30‐min intervals over 5 days. On the sixth day, the platform was removed, and the mice were tested for their ability to locate the former platform position, recording their paths and the number of crosses over the platform area within 60 s.

### 2.18 Statistical analysis

Statistical analysis was performed using Prism10.1.2 software (GraphPad Software, USA), and data presented as means±SD. Normality tests were performed first. Nonparametric tests were employed for data that did not follow a normal distribution. Comparisons between two groups were analyzed by independent two‐tailed Student's *t*‐tests, whereas those among more than two groups were analyzed using one‐way ANOVA with Newman–Keuls multiple comparison test. Statistical significance was set at *p* < 0.05.

## 3 RESULTS

### 3.1 Activated BV2(BV2^M1^) promoted HT22 inflammation and increased release of inflammatory factors

**FIGURE 1:**
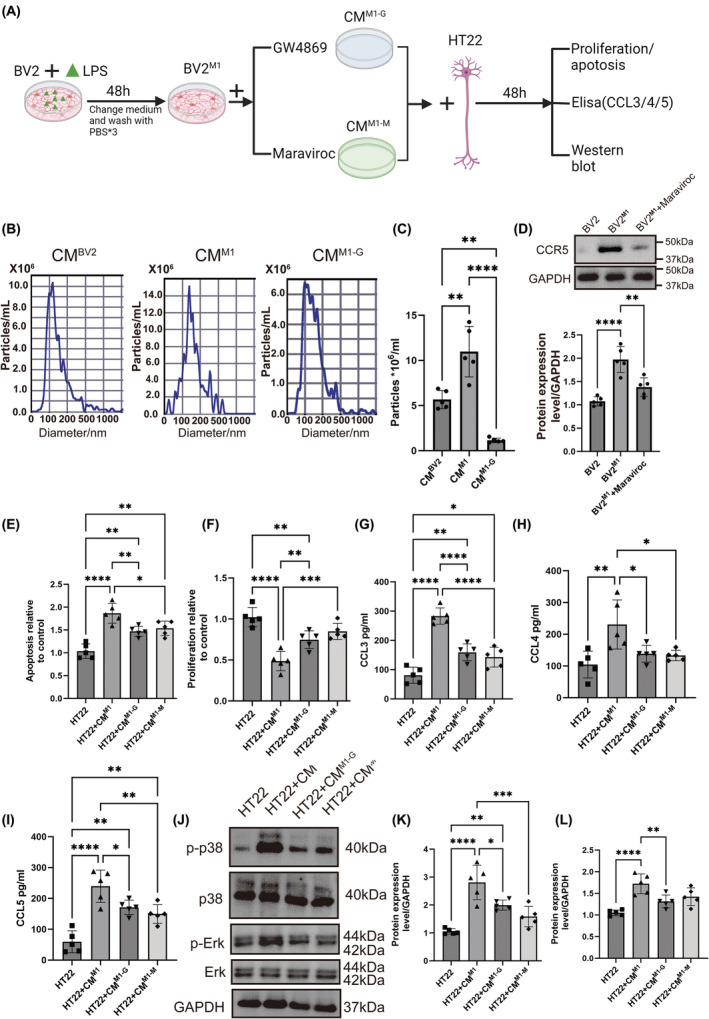
Activated BV2 (BV2^M1^) promoted HT22 inflammation and increases release of inflammatory factors. (A) BV2 cells were induced in vitro by applying LPS (2 μg/mL) for 48 h, the medium was changed, washed with PBS, and cultured for another 48 h. The conditional medium was harvested and supplemented with GW4869 (10 μM, extracellular vesicle inhibitor) or Maraviroc (200 nM), followed HT22 were cultured for 48 h. CM^BV2^ = conditional medium of BV2 without treatment; CM^M1^ = conditional medium of BV2^M1^; CM^M1‐G^ = conditional medium of BV2^M1^ supplemented with GW4869; CM^M1‐M^ = conditional medium of BV2^M1^ supplemented with Maraviroc. (B, C) Particle size distribution of EVs isolated from conditional medium of BV2 without treatment (CM^BV2^), conditional medium of BV2 treated with LPS (CM^M1^), and conditional medium of BV2 supplemented with GW4869 (CM^M1‐G^) was determined by NTA test. *n* = 5. (D) The protein expression levels of CCR5 in BV2, BV2^M1^, and BV2^M1+Maraviroc^ were analyzed using western blot. *n* = 5. (E) After 48 h incubation, apoptosis of HT22 was determined using an Apo‐ONE Homogeneous Caspase‐3/7 assay kit. (F) After 48 h incubation, proliferation of HT22 was determined using a BrdU–ELISA (enzyme‐linked immunosorbent assay)‐based assay. (G–I) The concentration of CCL3/CCL4/CCL5 within the conditional medium was determined by ELISA. (J–L) The phosphorylation levels of p38 and Erk were detected using western blot detection. All values represent mean ± standard deviation. *p < 0.05; **p < 0.01; ***p < 0.001; ****p < 0.0001; *n* = 5; One‐way ANOVA with Newman–Keuls multiple comparison test.

To verify whether BV2^M1^‐derived EVs (EVs^M1^) and CCR5 promote neuronal inflammation, we evaluated the effects of conditioned medium of LPS‐activated BV2 microglia (BV2^M1^) on HT22(#CL‐06697, Procell, China) neuronal cells. The resultant conditioned medium was treated with GW4869 (10 μM, inhibitor of EVs release, #D1692, sigma, USA) or Maraviroc (200 nM, CCR5 antagonist, #A8311, Apexbio, USA) to assess the influence of EVs inhibition and CCR5 blockade on HT22 neuronal cells (Figure 1A). We found that LPS promoted the EVs yield, and inhibited after GW4869 treatment (Figure 1B,C). The CCR5 expression in BV2^M1^ was significantly inhibited by Maraviroc (Figure 1D). As shown in Figure 1E,F, our results indicated significant increases in apoptosis and decreases in proliferation in HT22 cells cultured with the CM^M1^ (conditional medium of BV2^M1^), which were reversed by CM^M1‐G^ (conditional medium of BV2^M1^ supplemented with GW4869) and CM^M1‐M^ (conditional medium of BV2^M1^ supplemented with Maraviroc). Additionally, the levels of inflammatory cytokines CCL3, CCL4 and CCL5 were significantly higher in the conditioned medium, and these were partially reduced by both treatments of CM^M1‐G^ and CM^M1‐M^ (Figure 1G–I). Western blot analysis revealed increased phosphorylation of p38 and Erk signaling pathways, which were also moderately decreased in CM^M1‐G^ and CM^M1‐M^ groups (Figure 1J–L). These findings suggested that microglial activation contributes to neuronal inflammation and cell death, mediated by inflammatory cytokines and cellular stress pathways, which can be partially mitigated by targeting extracellular vesicles and CCR5 pathways.

### 3.2 Characterization and comparative analysis of control EVs and EVs^M1^

**FIGURE 2:**
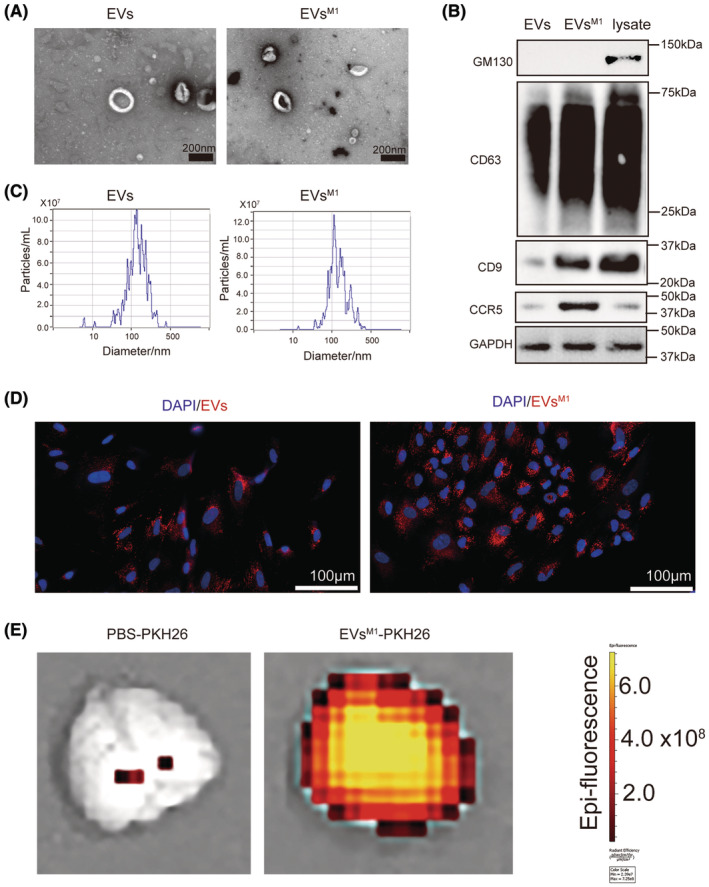
Characterization of control EVs and EVs^M1^. After stimulation with LPS (2 μg/mL) for 48 h, EVs^M1^ were isolated from supernatant of BV2 microglia. (A) TEM was used to analyze the morphology of control EVs and EVs^M1^; Scale bar: 100 nm. (B) Representative western blot image showing bands of standard positive EVs surface markers (CD81 and CD63), negative marker (GM130) and CCR5 of EVs, EVs^M1^, and BV2 cell lysate. *n* = 5. (C) Particle size distribution of control EVs and EVs^M1^ was measured by NTA. (D) PKH26‐labeled EVs/EVs^M1^ (red) were internalized by HT22 and visualized with fluorescent microscopy and cell nuclei were stained with DAPI (blue). Scale bar: 100 μm. *n* = 5. (E) Mice were stereotactically injected with PKH26‐labeled EVs. After 24 h, the brain was harvested and EVs^M1^‐PKH26 internalization was visualized by an IVIS imaging system.

In order to identify the role of EVs^M1^ in modulating neuronal inflammation, EVs were isolated from BV2 microglial cells under normal and LPS‐stimulated conditions (2 μg/mL for 48 h) for analysis. Transmission electron microscopy (TEM) revealed there was no obvious difference between control EVs and EVs^M1^. The images show that both types of EVs maintain typical vesicular structures and similar size (about 50–100 nm) (Figure 2A). Western blot analysis was performed to profile specific markers on the EVs. The blots for CD81 and CD63 confirmed the presence of these standard positive EV markers (CD63 and CD9) on both control EVs and EVs^M1^, in addition, the negative marker GM130 was absent in both EV samples, confirming the purity of the vesicle preparations. Notably, CCR5 was markedly upregulated in EVs^M1^, indicating a selective enrichment of this marker in EVs from BV2^M1^ (Figure 2B). Nanoparticle tracking analysis (NTA) showed that the particle size distribution of EVs^M1^ was similar to that of control EVs, with most particles measuring between 100 and 200 nm in diameter. This suggested that LPS stimulation does not significantly alter the size distribution of the extracellular vesicles released by BV2 cells (Figure 2C). Fluorescent microscopy was used to assess the internalization of PKH26‐labeled EVs and EVs^M1^ by HT22 cells. Both types of EVs were efficiently internalized, as evidenced by the red fluorescent labeling within the cells. Cell nuclei were counterstained with DAPI (blue), highlighting the uptake of red‐labeled EVs into the predominantly blue‐stained cell nuclei areas (Figure 2D). In vivo experiments involved stereotactic injection of PKH26‐labeled EVs into mice. After 24 h, brain tissues were harvested and analyzed for EV internalization, following visualized by an IVIS imaging system, demonstrating the potential of these vesicles for targeted delivery or interaction within the brain (Figure 2E). These results demonstrated the successful isolation and characterization of EVs and EVs^M1^ from BV2 microglia, highlighting significant differences in marker expression and internalization behavior that may reflect functional changes induced by LPS stimulation.

### 3.3 Impact of EVs produced from BV2^M1^ with CCR5 knockdown (EVs^M1‐CCR5^) on HT22

**FIGURE 3:**
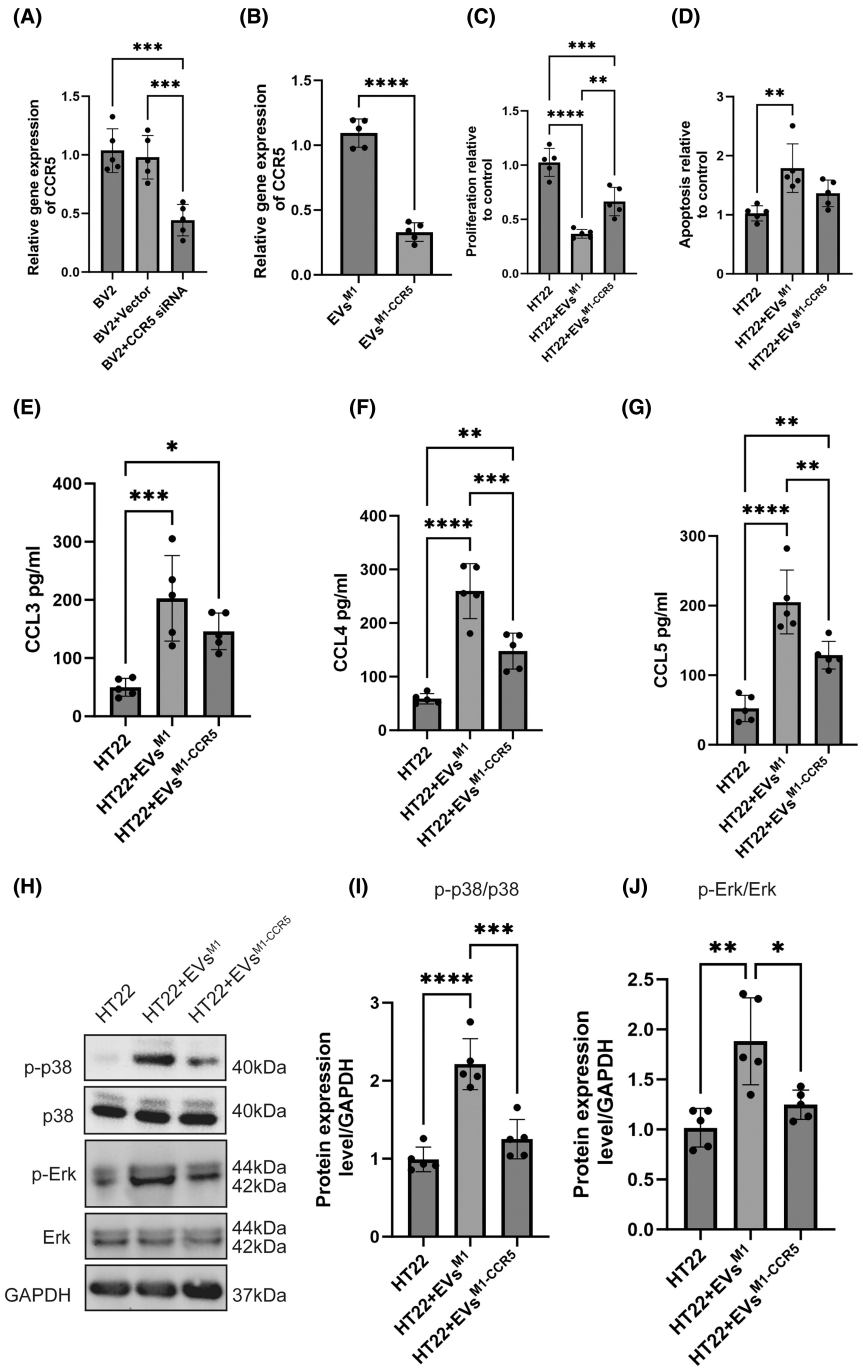
EVs derived from BV2^M1^‐CCR5 KO reversed the catabolic effect of EVs^M1^ on HT22. (A) Relative gene expression of CCR5 in BV2^M1^ and after vector, CCR5 siRNA treatments. *n* = 5; One‐way ANOVA with Newman–Keuls multiple comparison test. (B) Relative gene expression of CCR5 in control EVs^M1^ and EVs^M1‐CCR5^ was determined by qRT‐PCR. *n* = 5; independent two‐tailed Student's *t*‐tests. (C) Proliferation of HT22 treated with EVs^M1^ and EVs^M1‐CCR5^ was detected using a BrdU–ELISA‐based assay. *n* = 5; One‐way ANOVA with Newman–Keuls multiple comparison test. (D) Apoptosis of HT22 treated with EVs^M1^ and EVs^M1‐CCR5^ was determined using an Apo‐ONE Homogeneous Caspase‐3/7 assay kit. *n* = 5; One‐way ANOVA with Newman–Keuls multiple comparison test. (E–G) The concentration of CCL3/CCL4/CCL5 within the conditional medium was determined by ELISA. *n* = 5; One‐way ANOVA with Newman–Keuls multiple comparison test. (H–J) The phosphorylation levels of p38 and Erk were detected using western blot detection. *n* = 5; One‐way ANOVA with Newman–Keuls multiple comparison test. All values represent mean ± standard deviation. *p < 0.05; **p < 0.01; ***p < 0.001; and ****p < 0.0001.

Quantitative RT‐PCR analysis showed a significant reduction in the relative gene expression of CCR5 in EVs^M1‐CCR5^ compared to EVs^M1^, confirming successful knockdown of the CCR5 gene in the BV2^M1^ (Figure 3A) and EVs^M1‐CCR5^ (Figure 3B). The proliferation of HT22 cells treated with EVs^M1‐CCR5^ was notably higher than those treated with EVs^M1^, indicating that the absence of CCR5 in EVs^M1^ could mitigate the inhibitory effects on neuronal cell proliferation observed with EVs^M1^ (Figure 3C). Apoptosis assays revealed a decrease in caspase‐3/7 activity in HT22 cells treated with EVs^M1‐CCR5^ compared to those treated with EVs^M1^, suggesting that CCR5 on EVs^M1^ contributes to the proapoptotic signals in HT22 cells (Figure 3D). ELISA results showed that the concentrations of inflammatory cytokines such as CCL3, CCL4, and CCL5 in the conditioned medium of HT22 were lower in samples treated with EVs^M1‐CCR5^ than those treated with EVs^M1^. This indicated a reduced inflammatory signaling potential in the absence of CCR5 on EVs^M1^ (Figure 3E–G). In addition, western blot analysis of the phosphorylation levels of p38 and Erk revealed lower activation levels in HT22 cells treated with EVs^M1‐CCR5^ compared to those treated with standard EVs^M1^, highlighting a dampened stress and inflammation response due to the CCR5 knockdown (Figure 3H–J).

### 3.4 EVs^M1‐CCR5^ alleviated the inhibited neuronal protein expression induced by EVs^M1^ via CCR5/GPR/Ras axis

**FIGURE 4:**
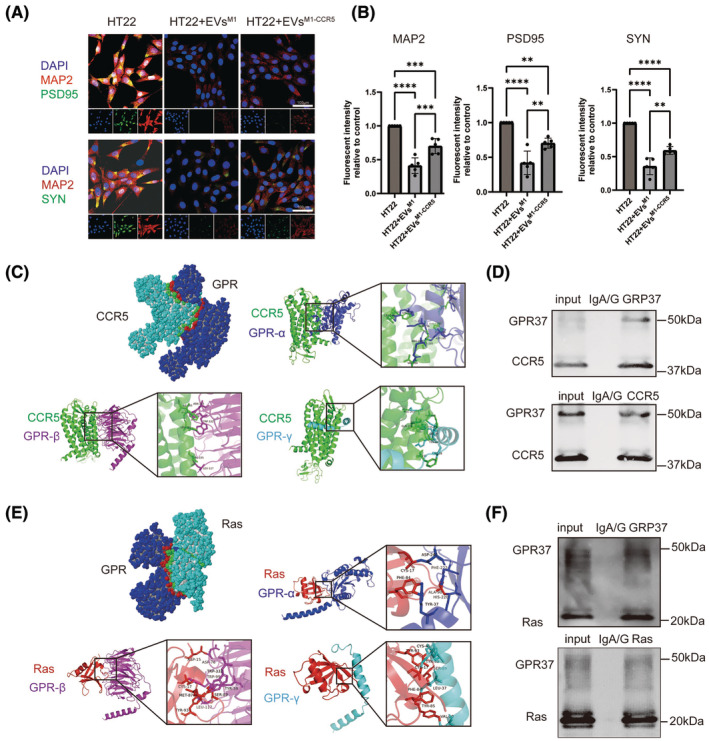
Effects of EVs^M1^ and EVs^M1‐CCR5^ on HT22 cells, and interaction analysis between CCR5, GPR, and Ras. (A) Immunofluorescence staining of HT22 cells treated with EVs^M1^ and EVs^M1‐CCR5^. Cells were stained for MAP2 (red), PSD95 (green), and SYN (green) with DAPI (blue) counterstaining for nuclei. Representative images are shown. (B) Quantitative analysis of immunofluorescence intensity for MAP2, PSD95, and SYN in HT22 cells treated with EVs^M1^, and EVs^M1‐CCR5^. All values represent mean ± standard deviation. *n* = 5; *p < 0.05; **p < 0.01; ***p < 0.001; and ****p < 0.0001; One‐way ANOVA with Newman–Keuls multiple comparison test. (C) Structure‐based protein interaction interface analysis of CCR5 with GPR proteins. Cartoon representations show interactions of CCR5 with GPRα, GPRβ, and GPRγ subunits. Enlarged views highlight the interaction interfaces. (D) Co‐immunoprecipitation (Co‐IP) experiments confirming the interaction between CCR5 and GPR37 proteins. Blots show the presence of GPR37 and CCR5 in input and immunoprecipitated samples. (E) Structure‐based protein interaction interface analysis of GPR with Ras proteins. Cartoon representations illustrate interactions of Ras with GPRα, GPRβ, and GPRγ. Enlarged views display the interaction interfaces. (F) Co‐immunoprecipitation (Co‐IP) experiments verifying the interaction between GPR proteins and Ras. Blots display the presence of GPR37 and Ras in input and immunoprecipitated samples.

Immunofluorescence staining was performed on HT22 cells treated with EVs^M1^ and EVs^M1‐CCR5^ to evaluate the expression of neuronal proteins MAP2, PSD95, and SYN (Figure 4A). Quantitative analysis revealed a significant decrease in the fluorescence intensity of MAP2, PSD95, and SYN in HT22 cells treated with EVs^M1^ compared to untreated cells (Figure 4B). This indicated that EVs^M1^ treatment inhibits the expression of these neuronal proteins. Conversely, treatment with EVs^M1‐CCR5^ significantly mitigated the inhibitory effects of EVs^M1^, as evidenced by the increased fluorescence intensity of MAP2, PSD95, and SYN compared to EVs^M1^‐treated cells. These results suggested that EVs^M1‐CCR5^ could alleviate the negative impact of EVs^M1^ on neuronal protein expression in HT22 cells. G‐protein‐coupled receptors (GPCRs) play a crucial role in the activation of the MAPK signaling pathway through their interaction with Ras proteins. To explore the potential interactions between CCR5 and GPR proteins, structure‐based protein interaction interface analysis was conducted. Docking models demonstrated that CCR5 interacts with all three subunits of GPR: GPRα, GPRβ, and GPRγ (Figure 4C). These interactions were visualized using cartoon representations, with enlarged views highlighting the specific interaction interfaces between CCR5 and each GPR subunit. Co‐IP experiments further confirmed these interactions. GPR37 and CCR5 were both detected in input and immunoprecipitated samples, indicating a physical association between these proteins in HT22 cells (Figure 4D). In addition to analyzing CCR5 and GPR interactions, the potential interactions between GPR proteins and Ras were also examined. Structure‐based protein interaction interface analysis revealed that Ras interacts with GPRα, GPRβ, and GPRγ subunits (Figure 4E). Co‐IP experiments confirmed the interaction between GPR proteins and Ras. Both GPR37 and Ras were present in the input and immunoprecipitated samples, indicating that GPR proteins associate with Ras in HT22 cells (Figure 4F).

### 3.5 Therapeutic effects of EVs^M1‐CCR5^ on synaptic plasticity and neuron development in a mouse model of POCD

**FIGURE 5:**
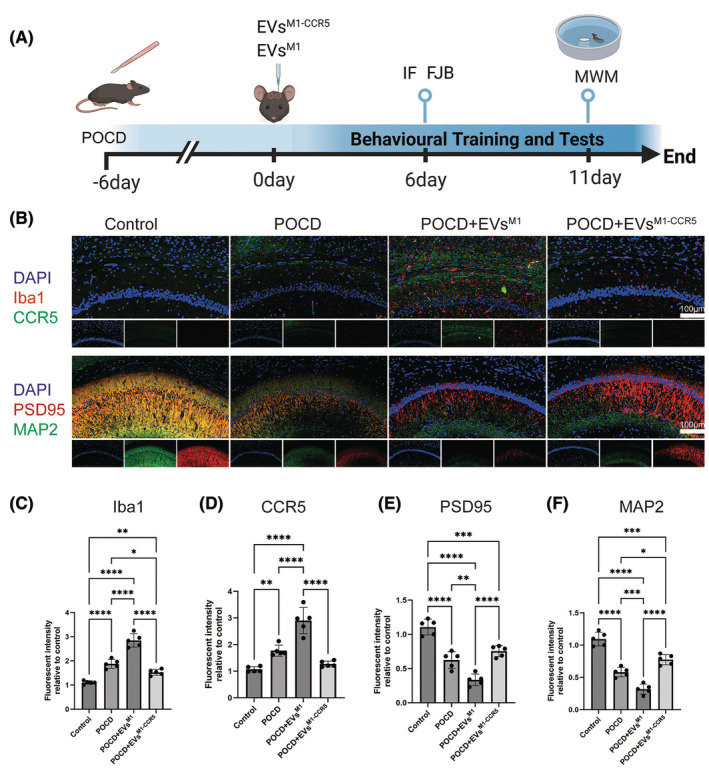
EVs^M1‐CCR5^ reversed the decrease of synaptic plasticity and neuron development induced by EVs^M1^ in a mice model of POCD. A mice model of POCD was established priority, after 6 days, POCD mice were stereotactically injected with EVs^M1^ or EVs^M1‐CCR5^ every 2 days. The immunofluorescence and FJB staining were performed on sixth day, and the MWM training was started from sixth day and tested on 11th day. (A) Schematic diagram. (B) The protein expression levels of Iba1 (M1 type microglia), CCR5, PSD95 (synaptic plasticity marker), and MAP2 (neuron development marker) were determined by immunofluorescence staining. (C–F) Quantitative analysis of the expression levels of Iba1, CCR5, PSD95, and MAP2. All values represent mean ± standard deviation. *n* = 5; *p < 0.05; **p < 0.01; ***p < 0.001; and ****p < 0.0001; One‐way ANOVA with Newman–Keuls multiple comparison test.

This study investigated the impact of EVs^M1^ and EVs^M1‐CCR5^ on a mouse model of postoperative cognitive dysfunction (POCD). Mice were subjected to stereotactic injections of EVs^M1^ and EVs^M1‐CCR5^ following the establishment of POCD. As depicted in the schematic, the mice were treated with EVs^M1^ or EVs^M1‐CCR5^ every 2 days starting from day 6 post‐POCD induction, with immunofluorescence (IF) and Fluoro‐Jade B (FJB) staining performed on day 6, followed by Morris Water Maze (MWM) testing from day 6 to day 11 (Figure 5A). Immunofluorescence staining was employed to assess the expression of microglial activation marker Iba1, the chemokine receptor CCR5, the synaptic plasticity marker PSD95, and the neuron development marker MAP2 in brain sections from control, POCD, POCD+EVs^M1^, and POCD+EVs^M1‐CCR5^‐treated mice. The staining revealed marked differences in protein expression among the groups (Figure 5B). Quantitative analysis showed a significant increase in Iba1 intensity in POCD mice, which was reduced in mice treated with EVs^M1‐CCR5^ compared to those treated with EVs^M1^, indicating a decrease in microglial activation due to the absence of CCR5 (Figure 5C). CCR5 expression was significantly higher in the POCD and POCD+EVs^M1^ groups compared to control. However, its expression was notably lower in the POCD+EVs^M1‐CCR5^ group, aligning with the knockdown of CCR5 in these vesicles (Figure 5D). The fluorescence intensity of PSD95 was decreased in POCD mice, indicative of reduced synaptic plasticity, which was further promoted in POCD+ EVs^M1^ group. This decrease was significantly mitigated in the POCD+EVs^M1‐CCR5^ group, suggesting that knocking down CCR5 in EVs may protect or restore synaptic functions disrupted by anesthesia and surgery (Figure 5E). Similarly, the expression of MAP2 was significantly decreased in POCD mice and POCD+EVs^M1^ group, reflecting impaired neuronal development induce by anesthesia and surgery, which was further exacerbated by EVs^M1^. Treatment with EVs^M1‐CCR5^ reversed this effect, indicating a beneficial role of CCR5 knockdown in promoting neuronal health in the context of POCD (Figure 5F).

### 3.6 Impact of EVs^M1‐CCR5^ on neuronal degeneration and inflammatory signaling pathways in a POCD model

**FIGURE 6:**
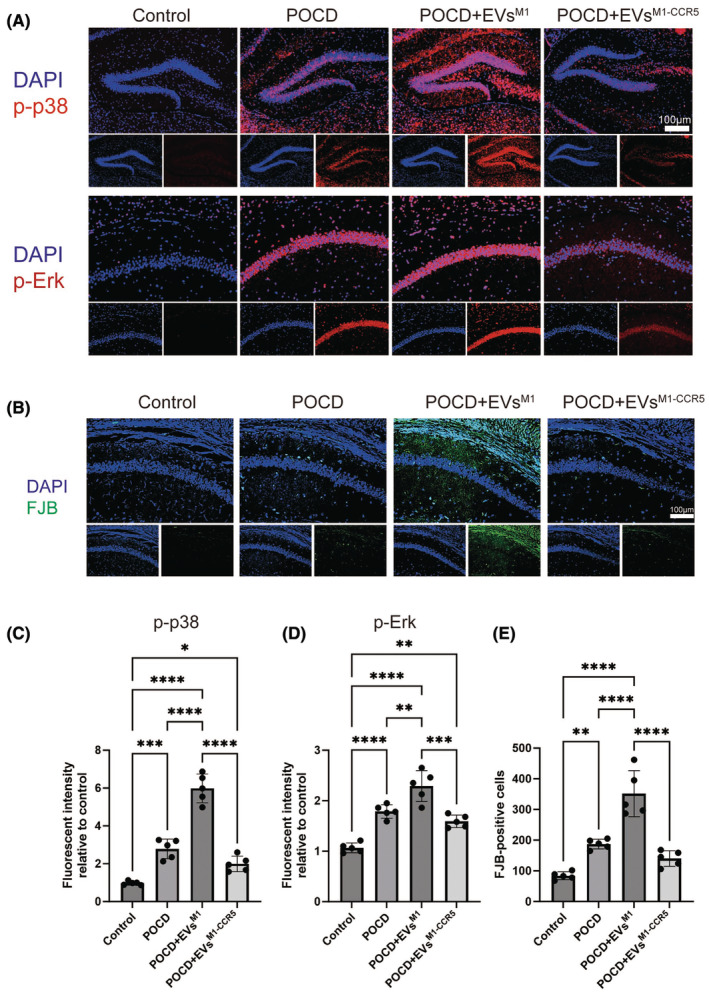
EVs^M1‐CCR5^ alleviated the phosphorylation levels of p38 and Erk, and ameliorated the neuron degeneration caused by EVs^M1^. (A) After treated with EVs^M1^ and EVs^M1‐CCR5^ for 6 days, the phosphorylation levels of p38 and Erk were detected using immunofluorescence. *n* = 5. (B) Degenerated neurons were detected using FJB staining. *n* = 5. (C, D) Quantitative analysis of the phosphorylation levels of p38 and Erk. (E) Quantitative analysis of degenerated neurons after different treatment. All values represent mean ± standard deviation. *n* = 5; *p < 0.05; **p < 0.01; ***p < 0.001; and ****p < 0.0001; One‐way ANOVA with Newman–Keuls multiple comparison test.

Immunofluorescence staining was utilized to examine the phosphorylation levels of p38 and Erk signaling pathways' key markers of cellular stress response pathways. After 6 days posttreatment of EVs^M1^ and EVs^M1‐CCR5^, the phosphorylation levels of both p38 and Erk were significantly elevated in POCD mice treated with EVs^M1^, indicating heightened neuronal inflammation. However, these changes were notably reduced in mice treated with EVs^M1‐CCR5^, suggesting a mitigating effect of the CCR5 knockdown on stress signaling (Figure 6A,C,D). Neuronal degeneration was assessed using FJB staining, a method for detecting degenerating neurons. FJB staining showed an increased number of degenerated neurons in POCD mice, which was exacerbated by treatment with EVs^M1^. Conversely, treatment with EVs^M1‐CCR5^ resulted in a marked reduction in FJB‐positive cells, indicating less neuronal degeneration (Figure 6B,E).

### 3.7 Restorative effects of EVs^M1‐CCR5^ on synaptic architecture and cognitive function in a POCD mouse model

**FIGURE 7:**
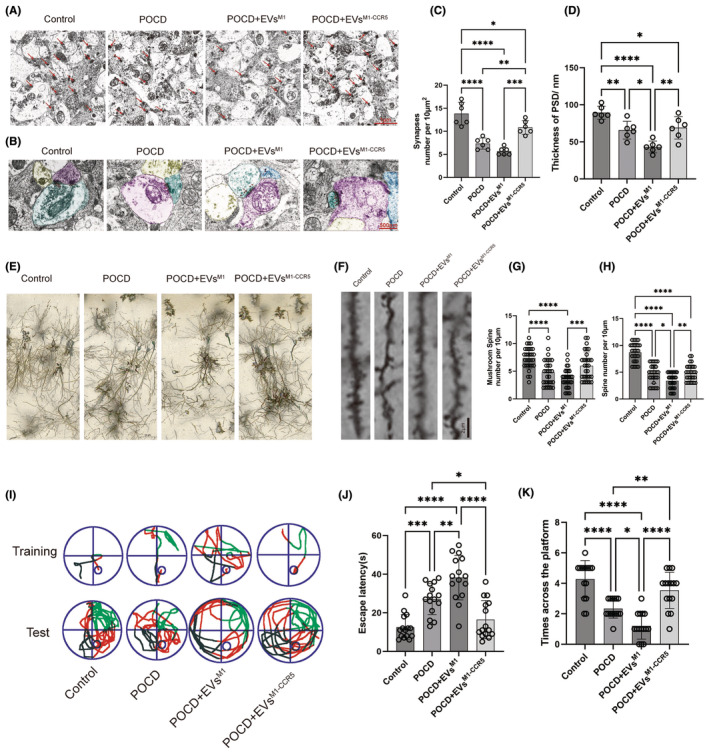
EVs^M1‐CCR5^ restored synaptic loss and memory defects in POCD model mice. (A) Representative images of hippocampal neurons. (B) Representative images of ultrastructure of synapse. (C) The number of synapses in hippocampal neuron, *n* = 6. (D) The thickness of postsynaptic density zone of synapse, *n* = 6. (E) Representative images of hippocampus via Golgi staining. (F–H) Representative dendritic spine and qualifications of spine and mushroom spine in POCD mice with EVs^M1^ and EVs^M1‐CCR5^ treatment. *n* = 30. (I) Representative swim tracks on the fifth day's training and test on the sixth day. (J) Average latency (time costing to find platform) per swim, *n* = 15; (K) Times spent across the platform in test swim. *n* = 15. *p < 0.05; **p < 0.01; ***p < 0.001; and ****p < 0.0001; One‐way ANOVA with Newman–Keuls multiple comparison test.

TEM was employed to investigate the intricate ultrastructure of neurons in the hippocampus. Microscopic images demonstrated a notable decrease of synapse in POCD and POCD+EVs^M1^ groups, which were reversed by POCD+EVs^M1‐CCR5^ (Figure 7A,C). The thickness of the postsynaptic density (PSD) consistently decreased substantially in POCD and POCD+EVs^M1^ groups, which was alleviated following EVs^M1‐CCR5^ treatment (Figure 7B,D). Golgi staining of hippocampal neurons provided visual evidence of the synaptic changes across different groups. Compared to the control group, POCD mice exhibited significant synaptic loss, which appeared aggravated in the POCD+EVs^M1^ group and more markedly restored in the POCD+EVs^M1‐CCR5^ group, suggesting a beneficial effect of the EVs^M1‐CCR5^ on synaptic preservation (Figure 7E). Representative imaging of dendritic spines showed that POCD induced a reduction in both total and mushroom spine density, which is indicative of synaptic damage. Treatment with EVs^M1^ resulted in a more decrease of spine density, while EVs^M1‐CCR5^ treatment led to a substantial restoration. This indicated that EVs^M1‐CCR5^ were effective in restoring synaptic structures affected by POCD and EVs^M1^ treatment (Figure 7F–H). Behavioral assessment through the Morris Water Maze (MWM) test illustrated the cognitive impact of treatments. Representative swim paths from the fifth day of training and the subsequent test day show that POCD mice had impaired navigation skills, which were aggravated by EVs^M1^, which was mitigated by EVs^M1‐CCR5^, as evidenced by more direct swim paths and fewer erroneous circles (Figure 7I). Quantitative data from the MWM revealed that the average latency to find the platform was significantly higher in POCD mice, especially in POCD+EVs^M1^, indicating memory impairment, which reduced in by EVs^M1‐CCR5^ treatment, suggesting EVs^M1‐CCR5^ enhanced memory retrieval and learning capabilities (Figure 7J). Furthermore, the number of times mice crossed the platform area during the test was higher in the EVs^M1‐CCR5^ group compared to both the POCD and POCD+EVs^M1^ groups, indicating improved spatial memory retention (Figure 7K). These results demonstrated that EVs^M1‐CCR5^ not only protect synaptic structures from POCD‐induced damage but also significantly improve cognitive functions, emphasizing the therapeutic potential of modifying extracellular vesicle properties in neurodegenerative and neuroinflammatory conditions.

## 4 DISCUSSION

The findings of this study deepen our understanding of the role of EVs^M1^ and CCR5‐mediated signaling in POCD. By examining the effects of modifying EVs^M1^ to lack CCR5 in a mouse model, we explored how the inflammatory properties of EVs^M1^ impact neuronal integrity and cognitive function. Our research sheds light on the intricate mechanisms of microglia–neuron interaction in the context of neuroinflammation.

Neuroinflammation plays an essential role in the development of POCD,^24^ which triggered by M1 type microglia.^25^ EVs have been reported that which regulated neuronal inflammation.^26^, ^27^ In addition, recent studies have found that CCR5 plays an important role in promoting inflammation in various CNS diseases.^28^, ^29^, ^30^ We were asked that what is the connection between EVs and CCR5 in POCD? Do they have a synergistic effect on the development of POCD? In in vitro experiments, we inhibited EVs secretion and CCR5 expression of BV2^M1^ by treated with GW4869 and Maraviroc, respectively. We found that the secretion of inflammatory factors and apoptosis of HT22 were significantly suppressed after being cultured with the above medium. We isolated EVs from BV2 without treatment (EVs) and LPS‐treated BV2 (EVs^M1^), then we were surprised to find that CCR5 was markedly upregulated in EVs^M1^. We hypothesized that BV2^M1^ promoted neuronal inflammation by delivering CCR5 via EVs^M1^.

We knock down CCR5 in BV2^M1^, isolated EVs^M1‐CCR5^, and treated HT22 in vitro. Our findings demonstrated that CCR5 plays a crucial role in mediating the effects of EVs^M1^ on neuronal cells, where its absence in EVs^M1‐CCR5^ leads to reduced apoptotic and inflammatory responses in HT22 cells. This suggests a potential therapeutic target in CCR5 for modulating microglial–neuronal interactions in neuroinflammatory contexts.^31^

Our immunofluorescence analysis showed a significant reduction in MAP2, PSD95, and SYN expression in HT22 cells treated with EVs^M1^, indicating that EVs^M1^ negatively impacts neuronal protein expression. These proteins are crucial for neuronal structure and synaptic function. The reduction of these proteins suggests that EVs^M1^ may contribute to neuroinflammatory conditions by disrupting neuronal integrity and synaptic connectivity. Interestingly, EVs^M1‐CCR5^ significantly mitigated the inhibitory effects on neuronal protein expression. CCR5 is a chemokine receptor involved in various immune responses, and its modulation has been linked to neuroprotective effects in various studies. The p38 MAPK pathway is particularly associated with neuroinflammatory responses and neuronal apoptosis. Activation of p38 MAPK in glial cells leads to the release of pro‐inflammatory cytokines and neurotoxic molecules, contributing to neuronal injury and degeneration.^32^ Sustained Erk activation impairs synaptic plasticity, a crucial process for learning and memory, by disrupting long‐term potentiation (LTP) and long‐term depression (LTD) mechanisms.^33^ Activation of the Ras‐MAPK pathway has been associated with neuroinflammation and neurodegenerative.^34^ Our structure‐based protein interaction interface analysis and co‐immunoprecipitation experiments revealed interactions between CCR5 and GPR subunits (GPRα, GPRβ, and GPRγ), as well as between Ras and GPR proteins. These findings suggest CCR5‐GPCRs‐Ras‐MAPK signaling pathway that could be involved in mediating the effects of EVs^M1^ on neuronal cells.

Microglia's activation is associated with neurodegeneration and neuroinflammation.^35^, ^36^ Our results suggested that EVs^M1‐CCR5^ could significantly mitigate microglial activation and improve markers of synaptic plasticity and neuron development that are adversely affected in a model of POCD, highlighting the potential therapeutic benefits of modifying extracellular vesicle properties to treat neuroinflammatory disease.

In this study, the administration of EVs^M1^ from LPS‐activated BV2 microglia exacerbated neuroinflammatory markers such as the phosphorylation of p38 and Erk, signaling molecules known to mediate inflammatory responses and neuronal stress pathways. These findings are consistent with previous studies that have linked the activation of these pathways with the progression of neuronal inflammatory diseases.^37^ In contrast, EVs^M1‐CCR5^, which lack the chemokine receptor CCR5, showed a reduced ability to activate these signaling pathways. The reduced phosphorylation levels of p38 and Erk in mice treated with EVs^M1‐CCR5^ suggested that CCR5 plays a critical role in mediating the inflammatory signals contained within EVs^M1^. These results are particularly significant considering the current understanding of CCR5 as a facilitator of neuroinflammation and its potential as a therapeutic target in neurodegenerative diseases.^38^

The synaptic and cognitive outcomes observed in our study further illustrate the functional implications of modulating CCR5 expression in EVs^M1^. Notably, EVs^M1‐CCR5^ treatment led to the preservation of synaptic markers PSD95, inhibited neurons degeneration, and improved the structural integrity of neuronal dendritic spines, which is essential for learning and memory processes.^39^, ^40^, ^41^ Behaviorally, the improvement in cognitive function, as evidenced by enhanced performance in the Morris Water Maze test in EVs^M1‐CCR5^‐treated mice, underscores the potential of targeting microglial‐derived EVs to mitigate cognitive decline. This improvement not only highlights the role of CCR5 in exacerbating cognitive deficits through inflammatory mechanisms but also supports the concept of using targeted EVs modifications as a strategy to influence brain health postoperatively.^42^

Our research demonstrated that EVs^M1‐CCR5^ significantly alleviate POCD and reduce neuroinflammation. Amantadine, an NMDA receptor antagonist, inhibits microglial activation and reduces neuroinflammation, showing efficacy in neurologic disorders.^43^, ^44^ Similarly, immunosuppressants such as rapamycin target the mTOR pathway to reduce pro‐inflammatory cytokine production, providing neuroprotection.^45^, ^46^ The targeted delivery of bioactive molecules by EVs^M1‐CCR5^ ensures localized modulation of microglial activity, enhancing therapeutic efficacy and reducing systemic side effects compared to traditional immunosuppressants. EVs^M1‐CCR5^ offer a promising and safer alternative, with the potential for personalized medicine by tailoring therapeutic molecules to individual patient needs.

The therapeutic implications of our findings suggest that targeting EV‐mediated pathways could offer a novel approach to managing POCD. By engineering microglial EVs to reduce their inflammatory potential through techniques like CCR5 knockdown or other molecular modifications, we can potentially harness these vesicles as vehicles for delivering anti‐inflammatory therapies directly to the CNS. The study primarily focused on the role of CCR5 in EVs^M1^. Although this approach provided significant insights, other chemokine receptors and signaling pathways might also play crucial roles in POCD and neuroinflammation, which were not explored in this research. Future research should explore other molecular targets and signaling pathways involved in microglial activation and neuroinflammation, beyond CCR5, to gain a comprehensive understanding of the mechanisms underlying POCD.

## 5 CONCLUSION

This study demonstrated that CCR5‐modified microglial EVs significantly influence neuroinflammatory signaling, neuronal integrity, and cognitive function in a mouse model of POCD. The findings highlight the pathophysiological roles of microglial EVs in POCD and suggest a new therapeutic approach potentially applicable to other neuroinflammatory or neurodegenerative diseases via the CCR5‐GPCRs‐Ras‐MAPK signaling pathway. Modulating EVs characteristics offers a promising strategy for developing more effective treatments for cognitive dysfunction associated with surgical procedures and beyond.

## AUTHOR CONTRIBUTIONS

ZQ and JLP: Contributed to sample collection, designed the methods, designed experiments design, performed validation, formal analysis, investigation, data curation, and writing (original draft, review, and editing); HTW and YS: Designed the methods, isolated EV, and performed NTA analysis; LW and LD: Immunofluorescence and section scanning; BC and YYZ: Western blot and animal experiments; QHX: Designed the methods, JJY: Conceptualization, writing (review and editing), project administration, and funding acquisition. All the authors have proofread the final version of the manuscript.

## FUNDING INFORMATION

This study was supported by the National Natural Science Foundation of China (81971020).

## CONFLICT OF INTEREST STATEMENT

There is no financial support or relationships that may pose conflict of interest and the authors declare no conflicts of interest.

## Data Availability

The data used to support the findings of this study are available from the corresponding author upon request.
