# Correction to “Modulating Neuroinflammation and Cognitive Function in Postoperative Cognitive Dysfunction via CCR5‐GPCRs‐Ras‐MAPK Pathway Targeting With Microglial EVs”

**DOI:** 10.1111/cns.70063

**Published:** 2024-09-27

Qi Z., Peng J., Wang H., et al., “Modulating Neuroinflammation and Cognitive Function in Postoperative Cognitive Dysfunction via CCR5‐GPCRs‐Ras‐MAPK Pathway Targeting With Microglial EVs,” *CNS Neuroscience and Therapeutics* 2024;30: e14924, https://doi.org/10.1111/cns.14924.

The funding number 81971020 in the Funding Information section is incorrect. This should be read as National Natural Science Foundation of China, Grant/Award Number: 82201331.

We apologize for this error.

